# Supplementary figures and images for: Efficacy of mesenchymal stromal cells in the treatment of unexplained recurrent spontaneous abortion in mice: An analytical and systematic review of meta-analyses
Source: PLoS One. 2023 Nov 27;18(11):e0294855. doi: 10.1371/journal.pone.0294855 (PMC10681256; doi:10.1371/journal.pone.0294855)

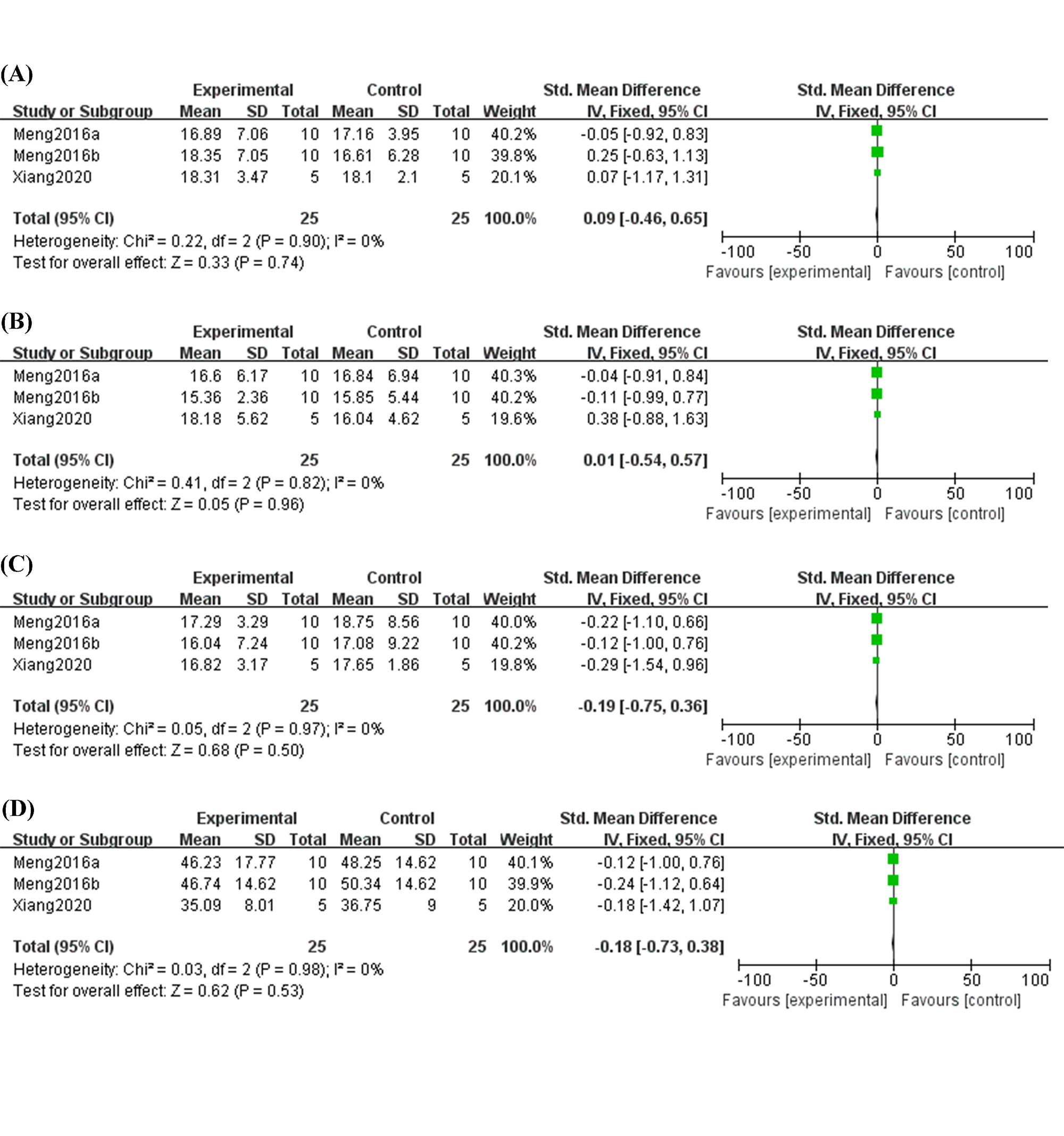

Supplement: S1 Fig — (TIF) [file pone.0294855.s001.tif]

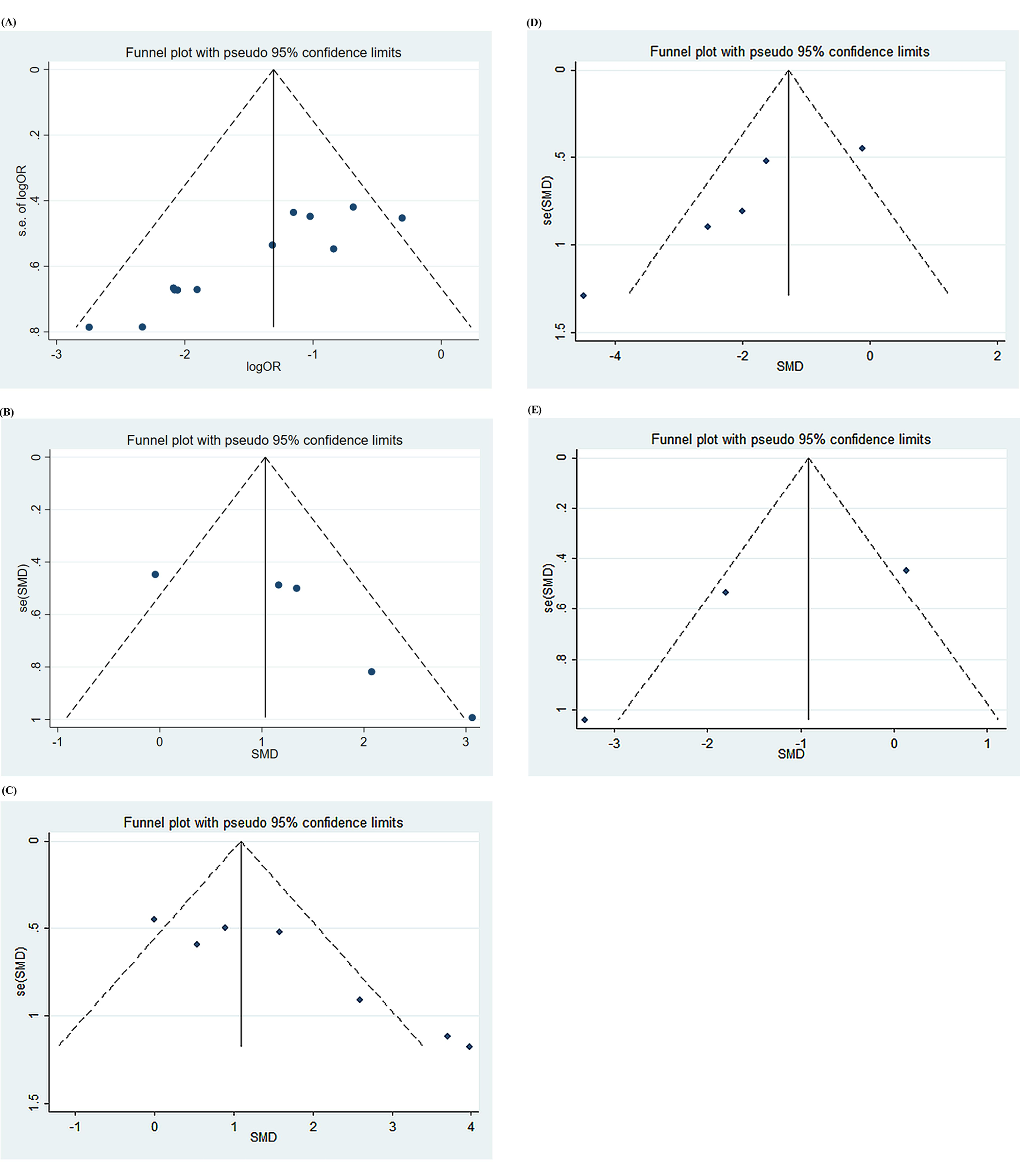

Supplement: S2 Fig — (TIF) [file pone.0294855.s002.tif]
