# Supplementary material for: Efficacy of mesenchymal stromal cells in the treatment of unexplained recurrent spontaneous abortion in mice: An analytical and systematic review of meta-analyses
Source: PLoS One. 2023 Nov 27;18(11):e0294855. doi: 10.1371/journal.pone.0294855 (PMC10681256; doi:10.1371/journal.pone.0294855)
Supplement: S2 Table — (PDF) [file pone.0294855.s004.pdf]

**Supplementary material 2: Table 2 Search strategy**

| Databases | Search terms | Number of records |
|---|---|---|
| Pubmed | #1 (("Abortion, Habitual"[Mesh]) OR ((((recurrent abortion[Title/Abstract]) OR (recurrent spontaneous abortion[Title/Abstract])) OR (RSA[Title/Abstract])))) OR (((unexplained recurrent spontaneous abortion[Title/Abstract]) OR (URSA[Title/Abstract])) OR (unexplained RSA[Title/Abstract]))<br>#2 ((("Exosomes"[Mesh]) OR (("Cell- and Tissue-Based Therapy"[Mesh]) OR (("Mesenchymal Stem Cells"[Mesh]) OR (((((((((MSCs[Title/Abstract]) OR (Bone mesenchymal stem cells[Title/Abstract])) OR (BMSCs[Title/Abstract])) OR (Adipose-derived stem cells[Title/Abstract])) OR (ADSCs[Title/Abstract])) OR (Umbilical cord mesenchymal stem cells[Title/Abstract])) OR (UCMSCs[Title/Abstract])) OR (Menstrual blood mesenchymal stem cells[Title/Abstract])) OR (MDMSCs[Title/Abstract]))))) OR (menstrual blood-derived stromal cells[Title/Abstract])) OR (MenSCs[Title/Abstract])<br>#1 AND #2 | 111 |
| Cochrane Library | (((((MeSH descriptor:[Abortion, Habitual] explode all trees) OR (recurrent abortion) OR (recurrent spontaneous abortion)) OR (RSA)))) OR (((unexplained recurrent spontaneous abortion) OR (URSA)) OR (unexplained RSA)) AND (((((((((((MeSH descriptor:[Cell- and Tissue-Based Therapy] explode all trees) OR (MeSH descriptor: [Mesenchymal Stem Cells] explode all trees) OR (MSCs) OR (Bone mesenchymal stem cells) OR (BMSCs) OR (Adipose-derived stem cells) OR (ADSCs) OR (Umbilical cord mesenchymal stem cells) OR (UCMSCs) OR (Menstrual blood mesenchymal stem cells) OR (MDMSCs) OR (menstrual blood-derived stromal cells) OR (MenSCs)) | 9 |
| EMBASE | #1 ('Abortion, Habitual'/exp) OR ('RSA') OR ('recurrent abortion') OR ('recurrent spontaneous abortion') OR ('unexplained recurrent spontaneous abortion') OR ('URSA') OR ('unexplained RSA') OR ('recurrent tubal abortion') OR ('habitual abortion')<br>#2 ((('Exosomes'/exp) OR (('Cell- and Tissue-Based Therapy'/exp) OR (('Mesenchymal Stem Cells'/exp) OR (((((((('MSCs') OR ('Bone mesenchymal stem cells')) OR ('BMSCs')) OR ('Adipose-derived stem cells')) OR ('ADSCs')) OR ('Umbilical cord mesenchymal stem cells')) OR ('UCMSCs')) OR ('Menstrual blood mesenchymal stem cells')) OR ('MDMSCs'))))) OR ('menstrual blood-derived stromal cells')) OR ('MenSCs')<br>#3 ('animal') OR ('animal experiment')<br>#1 AND #2 AND #3 | 30 |
| | | |
